# Ubiquitin-Specific Protease 18 in Chronic Kidney Disease—Another Emerging Biomarker to Consider?

**DOI:** 10.3390/biomedicines12051073

**Published:** 2024-05-13

**Authors:** Paulina Dziamałek-Macioszczyk, Agata Winiarska, Anna Pawłowska, Jan Macioszczyk, Paweł Wojtacha, Tomasz Stompór

**Affiliations:** 1 Department of Nephrology, Hypertension and Internal Medicine, School of Medicine, Collegium Medicum, University of Warmia and Mazury, 10-561 Olsztyn, Poland; 2 Research and Development Department, Visimind Ltd., 10-683 Olsztyn, Poland; 3 Department of Public Health, School of Health Sciences, Collegium Medicum, University of Warmia and Mazury, 10-082 Olsztyn, Poland

**Keywords:** ubiquitin-specific protease 18, deubiquitinase, cardiac remodeling, chronic heart failure, chronic kidney disease, proteinuria, albuminuria

## Abstract

Ubiquitin-specific protease 18 (USP18) is a protein recognized for its dual enzymatic and non-enzymatic nature. It is involved in many physiological processes like the cell cycle and cell signaling. It also suppresses heart muscle remodeling upon an increase in the afterload. The role of USP18 in kidney pathology remains unknown. The objective of the study was to assess the relationship between serum and urine USP18 levels, the factors contributing to cardiovascular risk, and the markers of kidney disease activity at different stages of chronic kidney disease (CKD). One hundred participants, aged between 24 and 85 years (mean 53.1 ± 17.1 years), were included. Five groups (n = 20 each) were recruited according to their renal status (healthy individuals, patients with proteinuric glomerulonephritis, patients with non-proteinuric CKD, patients who were treated with hemodialysis, and kidney transplant recipients). The measurements of serum and urine USP18 levels were performed using ELISA. The median serum USP18 level was the highest in healthy participants (1143.0 pg/mL) and kidney transplant recipients (856.6 pg/mL), whereas, in individuals with different forms of CKD, it fitted within the range of 402.1–471.9 pg/mL. Urinary USP18 reached the highest level in the group of CKD patients not yet on dialysis (303.3 pg/mL). Only in this group did it correlate with serum creatinine and urea concentrations. Our results suggest the inhibition of cardioprotective USP18 signaling when kidney function is impaired. Moreover, an increased level of urinary USP18 may indicate chronic tubular damage.

## 1. Introduction

Deubiquitinases (DUBs) are the wide group of enzymes which participate in the post-translational modification of proteins. They deconjugate ubiquitin and ubiquitin-like proteins (Ubls) from their substrates, thus influencing several biological processes, such as the cell cycle, cell division, and proliferation. Ubiquitin-specific proteases (USPs) are the largest family of proteins among DUBs. USPs comprise 54 members and one of the most intriguing is ubiquitin-specific protease 18 (USP18) [1]. USP18 is an intracellular cysteine protease highly expressed in the liver, spleen, and thymus. Its synthesis is stimulated by type I and type III interferons (IFNs), lipopolysaccharides of the bacterial wall, viral infections, the Toll-like receptor agonists, polyI:C (synthetic analogue of dsRNA), tumor necrosis factor alpha (TNF-α), and genotoxic stress [2,3]. The complex role of USP18 results from its dual nature. On the one hand, it executes an enzymatic function by removing the ubiquitin-like particle ISG15 (interferon-stimulated gene 15) from tagged proteins in a process called “deISGylation” and ubiquitin itself in deubiquitination. On the other hand, it plays non-enzymatic roles related to IFN inhibition by interacting with the IFNAR2 receptor and blocking the activation of the Janus-activated kinase/signal transducer and the activator of the transcription (JAK-STAT) signaling pathway [4,5,6]. Binding free ISG15 prevents USP18 from proteasomal degradation by the S-phase kinase-associated protein 2 (SKP2) and, therefore, is essential for stabilizing USP18 and providing the negative regulation of IFNs [7]. In some papers, USP18 is characterized as highly specific for ISG15, with no cross-reactivity towards ubiquitin [3,7].

USP18 participates in many physiologic and pathologic processes like the cell cycle, cell signaling, skeletal muscle cell differentiation, response to viral infections, carcinogenesis, and development of autoimmune diseases (including type 1 diabetes, systemic lupus erythematosus, or multiple sclerosis) [8,9,10,11]. USP18-deficient individuals die shortly after birth as a result of type-I IFN signaling dysregulation [7]. Mutations of the gene encoding USP18 lead to genetic disorders such as Di George and pseudo-TORCH syndromes [12].

Another role of USP18 is its contribution to the cardiac muscle remodeling and development of heart failure. Experiments performed in transgenic mice demonstrated that the increased USP18 expression in cardiomyocytes upon an increased afterload attenuated the cardiomyocyte hypertrophy and myocardial fibrosis, resulting in the delayed development of heart failure. The knock-out of the USP18 gene was associated with the increased pathological remodeling of the heart. USP18 turned out to be a modulator of the transforming growth factor beta-activated kinase 1 (TAK1)-p38-c-Jun N-terminal kinase 1/2 (JNK1/2) signaling pathway, essential in the remodeling process [4].

Several papers described the role of USP18 in cardiomyocyte damage, but its role in kidney injury remains unknown, though bidirectional cardio-renal interactions are well recognized in the pathophysiology of chronic kidney disease and heart failure. Acute or chronic kidney and heart function impairment constitutes cardiorenal syndrome (CRS) characterized by the escalating cascade of feedback mechanisms damaging both of these organs (perfectly co-operating in health). The complex pathophysiology of CRS remains not fully understood and consists of such processes as hemodynamic and neurohormonal disorders, activation of inflammation, atherosclerosis and fibrosis, or endothelial dysfunction [13,14,15]. The contribution of diet, abnormal microbiota composition, and fatty liver-associated renal disease (FACKD) should also be taken into account [16]. In hospitalized patients, CRS is associated with a worse prognosis, prolonged hospital stay, and increased risk of re-hospitalization and long-term mortality (up to 4 years of follow-up) [13]. Thus, the improvement of CRS diagnostic tools seems so important.

The goal of our study was to assess the association between serum and urine USP18 concentrations, cardiovascular risk factors, and different biomarkers of kidney disease in patients at different stages of chronic kidney disease (CKD), i.e., those with a biopsy-based diagnosis of glomerular pathology with proteinuria, those with CKD with an estimated glomerular filtration rate (eGFR) lower than 60 mL/min/1.73 m^2^, patients after kidney transplantation, and those treated with hemodialysis because of end-stage renal disease (ESRD).

## 2. Materials and Methods

### 2.1. Study Population

One hundred men were enrolled to our project. The mean age of the participants equaled 53.1 ± 17.1 years (range between 24 and 85 years), and 46% were female. The participants were divided into five groups, each consisting of 20 individuals. Similar study group has been defined in our previous paper on Dickkopf 3 protein in chronic kidney disease [17].

Group 1 (G1) served as the control and comprised healthy participants with normal renal function, i.e., eGFR greater than or equal to 60 mL/min/1.73 m^2^, and normal urine biochemistry and sediment. None of these individuals ever suffered from any chronic diseases, along with arterial hypertension (AHT) or diabetes (DM).

Group 2 (G2) included patients with normal kidney function who met the criteria to perform diagnostic kidney biopsy. The enrollment conditions for this group were eGFR greater than or equal to 60 mL/min/1.73 m^2^ and urinary albumin-to-creatinine ratio (UACR) greater than 0.5 g/g and/or abnormalities in urinary sediment. Individuals with diagnosis of DM, history of immunosuppressive therapy, or cardiovascular events were excluded after screening 50 subsequent patients. Diagnoses based on the kidney biopsy results in this group were IgA nephropathy (40%; 8 patients), anti-PLA2R antibody-positive membranous nephropathy (25%; 5 patients), primary focal/segmental glomerulosclerosis (15%; 3 patients), type IV collagen disease (10%; 2 patients), minimal change disease, and C3 nephropathy with a membrano-proliferative pattern of injury (5% each; 1 patient).

The third group (G3) consisted of patients with CKD at stages G3–G5 (in case of G5, those who were not on renal replacement therapy), who were recruited 3.5 ± 7.8 years after CKD was diagnosed. All participants were patients from our ambulatory care department. Four individuals (20%) had a previous cardiovascular event, but none of them were diagnosed with DM or underwent immunosuppressive treatment at the time of enrollment. The origin of CKD in this group was ischemic/hypertensive nephropathy or cardio-renal syndrome (60%; 13 patients), chronic glomerulonephritis (15%; 3 patients), nephrolithiasis, autosomal dominant polycystic kidney disease (ADPKD), and CKD following chronic pyelonephritis (5% each; 1 patient).

Patients in the fourth group (G4) were receiving dialysis treatment in our hemodialysis center, with a mean time on hemodialysis equaling 4.1 ± 7.2 years. All of them underwent three dialysis sessions weekly for 4.0–4.5 h using High Flux biocompatible FX-class dialyzers (Fresenius, Bad Homburg, Germany). Among this group, six individuals had a prior history of cardiovascular incidents; none of them received immunosuppressive agents at the time of enrollment. The causes of ESRD in this group were as follows: glomerulonephritis (55%; 11 patients), ischemic/hypertensive nephropathy (20%; 4 patients), and ADPKD (5%; 1 patient). The origin of ESRD remained unknown in terms of 4 patients (20%).

The fifth group (G5) consisted of individuals after kidney transplantation (KTx) with an eGFR greater than or equal to 60 mL/min/1.73 m^2^. Mean time following KTx equaled 3.7 ± 5.4 years. Mean time on renal replacement therapy before KTx was 2.6 ± 1.5 years. One patient (5%) had a previous cardiovascular event. Immunosuppressive treatment in this group included steroids + mycophenolate mofetil + tacrolimus (70%), steroids + mycophenolate mofetil + cyclosporine (25%), and steroids + mycophenolate sodium + tacrolimus (5%).

### 2.2. Laboratory Tests and Cardiovascular Status Evaluation

Fasting blood specimens were taken from every individual (in G4 on the day of dialysis before the procedure). First, void urine specimens were not obtained from participants in G4 due to either anuria or minimal urine output. The biochemical markers were assessed at a licensed laboratory by the Cobas 6000 analyzer (Roche, Basel, Switzerland). UACR was calculated using urine albumin, which was measured by an immunoturbidimetric assay conducted by the same analyzer. Heart rate (HR) and blood pressure (BP) were evaluated by the Omron M3 device (Kyoto, Japan), following the ESH/ESC Guidelines. The Sphygmocor X-cell device (AtCor Medical Pty. Ltd., Sydney, Australia) was used to determine a pulse wave velocity (PWV).

### 2.3. USP18 Assessment

Blood samples collected after fasting used to assess USP18 were immediately centrifuged after collection at 4 °C with a speed of 1000× *g* for 15 to 30 min. Every serum and urine specimen was subsequently preserved at −80 °C. Prior to analysis, the samples were thawed at −20 °C for a day, and then at 6 °C for a next day, and, eventually, at ambient temperature for 30 min, followed by blending using a vortex mixer. The USP18 levels were assayed using the Human Ubiquitin Specific Peptidase 18 ELISA Kit (Cat. No. E4837Hu) provided by Bioassay Technology Laboratory (Shanghai, China). To ensure accuracy, all tests for each sample were performed twice, resulting in a value of coefficient of variation (CV) of 0.84%. It was computed as the proportion of the standard deviation to the mean, centuplicated. To account for variations in urine volume, urinary USP18 (uUSP18) concentrations were normalized to urinary creatinine levels, yielding the uUSP18/creat. ratio.

### 2.4. Definitions

Body mass index (BMI) was determined by dividing an individual’s weight in kilograms by the square of their height in meters. AHT was defined as systolic blood pressure (SBP) equal to or greater than 140 mmHg and/or a diastolic blood pressure equal to or greater than 90 mmHg and/or being on any antihypertensive therapy, in accordance with the 2019 ESH/ESC Guidelines. The eGFR was assessed using the abbreviated MDRD formula. A cardiovascular incident was described as a previous myocardial infarction and/or stroke, a transient ischemic attack, peripheral vascular disease, or diagnosis of heart failure. The latter definition was grounded on clinical criteria and echocardiography result and/or the measurement of NT-pro-BNP levels. The diagnosis of chronic kidney disease was based on and staged following the KDIGO criteria.

### 2.5. Statistical Investigation

Statistical investigation was conducted using the SciPy library (version 1.7.1) in the Python (version 3.8.10, Python Software Foundation, Beaverton, OR, USA). Shapiro–Wilk test was used to verify if data are normally or non-normally distributed (test of normality). Data distribution determined if parametric or nonparametric tests were used to check differences between the groups. Data were non-normally distributed; therefore, Kruskal–Wallis test was used. If the test result indicated differences between the groups, Dunn’s test with Bonferroni correction was applied to make pairwise comparisons. Spearman’s correlation coefficients were computed to establish the relationship between USP18 and biochemical parameters. USP18 protein quantities in serum and urine were demonstrated as the median and interquartile range (IQR). A value of *p* less than 0.05 was judged statistically significant.

## 3. Results

**Figure 1:**
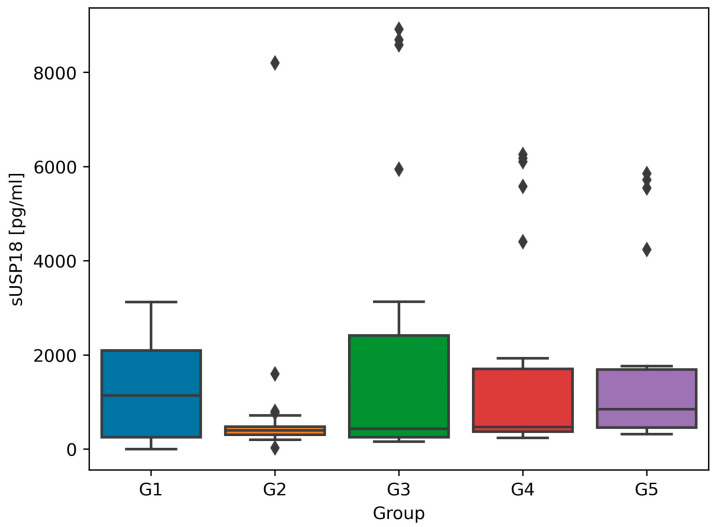
Median and interquartile ranges for serum ubiquitin-specific protease 18 (sUSP18) values in all study groups. The rhomboids represent outliers.

The serum USP18 (sUSP18), uUSP18, and uUSP18/creat. distribution in all groups was non-normal. Table 1 presents the anthropometric and biochemical parameters across all the groups and results of Kruskal–Wallis tests. The highest median sUSP18 concentration was found in G1—healthy individuals (1143.0 pg/mL, IQR 253.1–2095.6), followed by G5—kidney transplant recipients (853.6 pg/mL, IQR 457.8–1694.1). In other groups, the median values of sUSP18 were comparable, i.e., 402.1 pg/mL (IQR 318.6–719.4) in G2, 426.2 pg/mL (IQR 236.5–2895.5) in G3, and 471.9 pg/mL (374.9–1706.8) in G4 (Figure 1).

The null hypothesis for the Kruskal–Wallis test (no differences among all groups) was confirmed for DBP (*p* = 0.068), HR (*p* = 0.596), sUSP18 (*p* = 0.24), and uUSP18/creat. (*p* = 0.65). For other parameters, at least one of the groups was statistically different from the rest. We found an inverse correlation between sUSP18 and cardiac biomarkers in G2 (troponin T with r = −0.49, *p* = 0.0013 and NT-pro-BNP with r = −0.38, *p* = 0.011). In G4, sUSP18 was correlated with total cholesterol (r = 0.42, *p* = 0.0084). In G5, correlations of sUSP18 with BMI (r = 0.51, *p* < 0.001) and HR (r = 0.43, *p* = 0.0038) and (inverse) with urea concentration (r = −0.45, *p* = 0.0012) were found.

**Figure 2:**
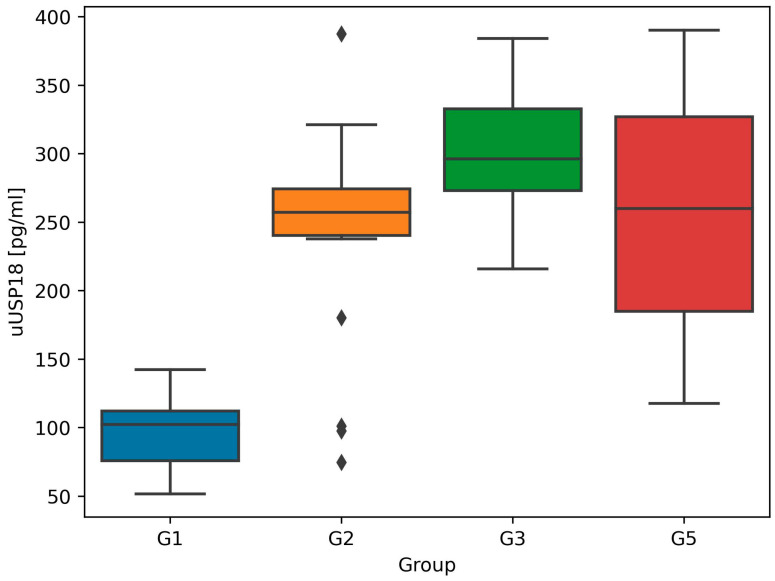
Median and interquartile ranges for urinary USP18 (uUSP18) values in all study groups. The rhomboids represent outliers. In G4, uUSP18 was not measured because of negligible urine volume.

**Figure 3:**
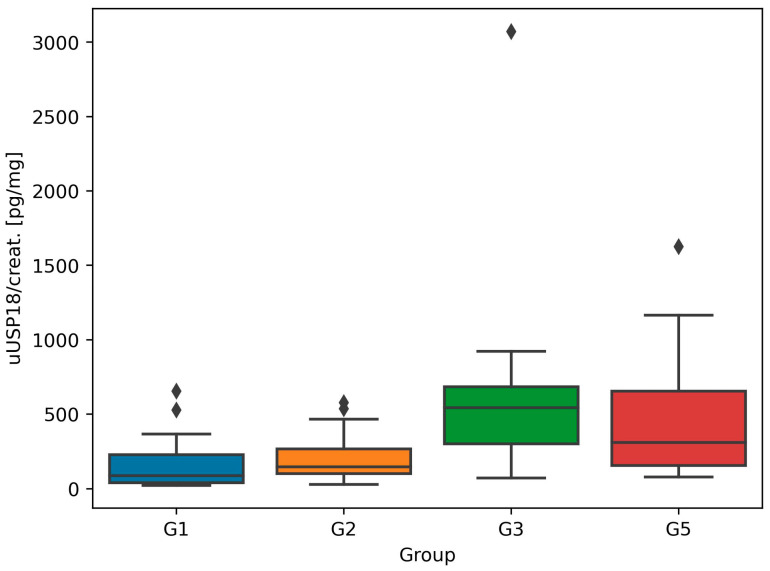
Median and interquartile ranges for urinary USP18 normalized to urinary creatinine (uUSP18/creat.) values in all study groups. The rhomboids represent outliers. In G4, uUSP18/creat. was not measured because of negligible urine volume.

The highest uUSP18 concentration was found in G3—the group of patients with advanced CKD not yet on renal replacement therapy (303.3 pg/mL, IQR 283.6–346.0). Study participants with normal kidney function (eGFR ≥ 60 mL/min) were characterized by lower median uUSP18 values. In G2, it equaled 256.8 pg/mL (IQR 237.9–274.1) and, in G5, 259.9 pg/mL (185.0–327.0), respectively. The lowest uUSP18 was measured in healthy individuals of G1 and equaled 102.2 pg/mL (75.7–112.1) (Figure 2). The adjustment to urinary creatinine levels confirmed these differences (Figure 3).

**Figure 4:**
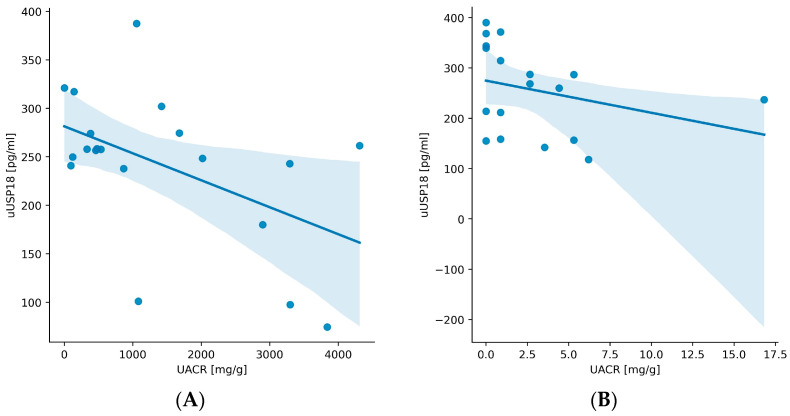

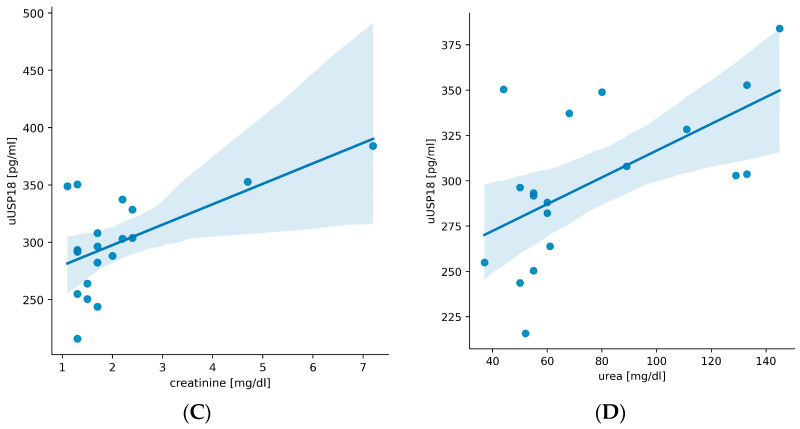
Spearman’s correlation analysis between uUSP18 and different parameters: (**A**) correlation between uUSP18 and urinary albumin-to-creatinine ratio (UACR) in group 2, (**B**) correlation between uUSP18 and UACR in group 5, (**C**) correlation between uUSP18 and creatinine in group 3, and (**D**) correlation between uUSP18 and urea in group 3. The blue lines illustrate the linear fit of data, the blue area represents the confidence interval.

The Kruskal–Wallis test revealed significant differences in uUSP18 between the groups (*p* < 0.001). To determine the exact source of this difference, Dunn’s test with the Bonferroni adjustment was used to make pairwise comparisons. It unveiled significant differences when G1 was compared with G2 (*p* = 0.0029), G3 (*p* < 0.001), G4 (*p* < 0.001), and G5 (*p* < 0.001). In G1, uUSP18 was correlated with DBP (r = 0.42, *p* = 0.032), PWV (r = 0.43, *p* = 0.022), creatinine (r = 0.47, *p* = 0.045), and troponin T (r = 0.50, *p* < 0.001). In G2, we found an inverse correlation between uUSP18 and UACR (r = −0.56, *p* = 0.0082) (Figure 4A). The same inverse correlation was found in G5 (r = −0.41, *p* = 0.0076) (Figure 4B). Moreover, uUSP18 was correlated with the BMI in G5 (r = 0.58, *p* < 0.001) and G3 (r = 0.47, *p* = 0.0038). In G3, we also observed the correlation with creatinine (r = 0.52, *p* = 0.0033) (Figure 4C), urea (r = 0.58, *p* = 0.0039) (Figure 4D) and troponin T (r = 0.44, *p* < 0.001).

## 4. Discussion

The heart and kidneys affect each other in multiple ways. The complex process of the mutual impairment of heart and kidney function is hemodynamic, hormonal, and inflammatory in nature [13,14,15]. USP18 plays a well-described protective role in the development of heart failure but its role in kidney injury remains unrecognized.

Both in murine experimental models and in humans, USP18 inhibits myocardial hypertrophy via the TAK1-p38-JNK1/2 axis upon an increased afterload [4]. In our earlier work, we assessed sUSP18 in men with AHT yet naïve to treatment and those without AHT, and we confirmed the role of this protein as an indicator of an enhanced afterload. Based on the observation that systolic–diastolic hypertensive (otherwise healthy) subjects were characterized by significantly higher median sUSP18 values than other groups (diastolic hypertensive group and healthy individuals), we hypothesized that high serum USP18 concentrations may correspond with a counterregulatory process aiming to compensate for cardiomyocyte contractile function and stabilize their structure [18]. In our current study, healthy individuals in G1 (using no medications, never diagnosed with AHT, cardiovascular, or any other chronic disease) and KTx patients in G5 (with treated AHT and excellent graft function) were characterized with the highest sUSP18 concentrations, with median values of 1143 pg/mL and 853.6 pg/mL, respectively (Figure 1). Patients from G2–G4 also suffered from AHT and were treated with antihypertensive drugs; nevertheless, sUSP18 levels in these groups were lower (402.1–471.9 pg/mL). This intriguing observation may imply that, when kidney function is impaired, the synthesis of USP18 is suppressed and it may no longer evoke its cardioprotective effect. In such a case, kidney transplantation seems to reverse this effect and ameliorate USP18 synthesis.

Based on our best knowledge, other research assessing uUSP18 concentrations in humans with cardiovascular diseases or CKD have not yet been published. Our study revealed that median uUSP18 was the highest in G3—in patients with a considerably reduced eGFR (<60 mL/min, mean eGFR 32.8 mL/min). In groups with preserved kidney function and eGFR ≥ 60 mL/min (G1, G2 and G5), these values were significantly lower (Figure 2 and Figure 3). As we observed the opposite tendency for sUSP18 (sUSP18 was the highest in G1), the possible explanation of this phenomenon is the impaired tubular reabsorption or increased tubular secretion of USP18 in the damaged kidney. Furthermore, in G3, uUSP18 correlated with serum creatinine (Figure 4C) and urea (Figure 4D), which suggests the potential role of uUSP18 as a biomarker of chronic kidney injury. As biomarkers of chronic tubular injury are lacking, it would be a significant step in the development of kidney diseases diagnostic methods, especially if a biomarker is present in urine and collecting blood is not necessary.

UACR is a parameter commonly used in everyday practice in assessing endothelial dysfunction [19,20]. UACR inversely correlated with uUSP18 in G2 (glomerulonephritis group, median UACR 960.6 mg/g, IQR 369.3–2238.1) (Figure 4A) and G5 (KTx group, median UACR 3.5 mg/g, IQR 1.3–5.3) (Figure 4B). Although the molecular weight of USP18 is lower than albumin (43 vs. 69 kDa) [1,2], the degree of their glomerular filtration is likely similar; hence, we hypothesize that markedly increased uUSP18 and uUSP18/creat. in patients with injured kidneys may result from an altered tubular handling which affects its final excretion. An additional investigation is required to identify variables that influence the glomerular filtration and tubular reabsorption of these proteins. The observation made in KTx patients is intriguing: sUSP18 is comparable to healthy controls, whereas the uUSP18 excretion remains high (which may suggest ongoing injury to renal tubules despite preserved GFR; Figure 1, Figure 2 and Figure 3). This observation may also point towards USP18 as a promising biomarker indicating renal (tubular) injury.

Obesity is one of the most common risk factors of cardiovascular disease [21]. Urinary USP18 was correlated with the BMI in G3 and G5 (CKD and KTx group), and with troponin T in G3. Further studies are needed to confirm the association of uUSP18 with cardiovascular risk factors.

Our study has certain limitations. One notable constraint is its cross-sectional nature, lacking a follow-up. Another is the fact that the assessment of cardiovascular status relied solely on medical history, cardiac biomarkers, and PWV, and an echocardiography evaluation was available for only a limited number of patients. The study group with a total of 100 participants, divided into 20 subjects each, is relatively small. Nevertheless, this study represents the first comprehensive insight into the role of serum and urine USP18 in the pathophysiology of the entire spectrum of renal disease. Furthermore, it is essential to highlight that a precise (biopsy-based) diagnosis of chronic kidney disease (CKD) was established for all patients in the G2 group, and for the majority of subjects in the G5 group.

## 5. Conclusions

Serum USP18 concentrations were the highest in healthy individuals and kidney transplant recipients. In other participants with CKD, they were considerably lower, which suggests that, in CKD, the cardioprotective effect of USP18 is suppressed. Kidney transplantation seems to reverse this effect. In contrast, the uUSP18 level was the highest in the group of participants with advanced CKD and only in this group did it correlate with creatine and urea concentrations. We hypothesize that this phenomenon may be a result of altered USP18 tubular reabsorption or secretion and uUSP18 might be a marker of chronic tubular/interstitial injury.

Additional research is needed to fully understand the role of USP18 in kidney diseases. Sadly, despite the rapidly growing number of various biomarkers of kidney pathologies, like neutrophil gelatinase-associated lipocalin (NGAL) or kidney injury molecule 1 (KIM-1), they rarely become useful in clinical practice. The serum creatinine level and albuminuria are still the diagnostic gold standard of renal function impairment.

## Figures and Tables

**Table 1:** Presentation of anthropometric and biochemical parameters in all study groups (*n* = 20). Respecting the data distribution, mean and standard deviation were calculated for parameters with normal distribution. Median and interquartile range were calculated for parameters with non-normal distribution. *p*-values were calculated for 5 independent groups by Kruskal–Wallis test.

Parameter	Unit	G1	G2	G3	G4	G5	*p*-Value
Age	[years]	30.5 (28.0; 38.0)	56.0 (39.0; 63.0)	69.1 ± 11.0	59.3 ± 14.3	50.5 ± 12.3	<0.001
Weight	[kg]	71.0 ± 13.6	83.7 ± 12.5	82.5 ± 15.4	75.9 ±12.7	77.4 ± 12.8	0.026
BMI	[kg/m^2^]	24.3 ± 3.7	27.0 ± 4.0	28.7 (26.4; 31.2)	26.1 (23.9; 28.5)	26.9 ± 3.2	0.003
SBP	[mmHg]	122 ± 8	141 ± 20	144 ± 17	145 ± 20	133 ± 15	<0.001
DBP	[mmHg]	77 (70; 85)	86 ± 13	84 ± 12	81 ± 12	83 (80; 90)	0.068
HR	[beats/min]	75.4 ± 10.1	78.0 (70.0; 81.0)	75.7 ± 13.2	78.0 (76.0; 80.0)	76.8 ± 7.9	0.596
PWV	[m/s]	7.57 ± 1.27	8.54 ± 1.80	11.56 ± 1.77	8.30 (7.65; 9.45)	9.41 ± 2.13	<0.001
Total cholesterol	[mg/dL]	179 ± 28	212 (184; 259)	169 ± 40	148 ± 29	171 ± 33	<0.001
LDL	[mg/dL]	104 ± 32	143 (112; 178)	102 ± 27	87 ± 29	84 ± 23	<0.001
HDL	[mg/dL]	64 ± 17	56 (46; 67)	52 ± 12	45 ± 16	65 ± 19	0.005
Triglycerides	[mg/dL]	101 ± 38	160 (94; 184)	138 (112; 191)	168 ± 71	139 (127; 169)	0.003
Creatinine	[mg/dL]	0.85 ± 0.11	0.90 (0.80; 1.00)	1.70 (1.30; 2.25)	8.22 ± 2.71	1.00 (0.90; 1.03)	<0.001
eGFR	[ml/min]	86.7 (79.0; 97.5)	85.7 ± 23.7	32.8 ± 11.3	6.3 (4.7; 8.3)	69.4 (62.6; 82.9)	<0.001
Urea	[mg/dL]	26.9 ± 6.2	36.2 ± 11.5	60.5 (54.3; 94.5)	118.1 ± 36.5	36.5 ± 7.5	<0.001
UACR	[mg/g]	2.7 (1.3; 2.7)	960.6 (369.3; 2238.1)	13.3 (3.5; 556.6)	-	3.5 (1.3; 5.3)	<0.001
Troponin T	[ng/mL]	0.0040 (0.0030; 0.0043)	0.0080 (0.0048; 0.014)	0.016 (0.009; 0.033)	0.053 (0.036; 0.083)	0.009 (0.007; 0.014)	<0.001
CK-MB	[U/L]	12.5 (11.0; 16.3)	14.0 (12.0; 17.0)	14.0 (12.0; 18.3)	11.58 ± 3.20	17.6 ± 5.6	0.002
NT-proBNP	[pg/mL]	40 ± 19	95 (38; 261)	457 (237; 2896)	6420 (3317; 14187)	141 (107; 164)	<0.001
sUSP18	[pg/mL]	1143.0 (253.1; 2095.6)	402.1 (318.6; 719.4)	426.2 (236.5; 2895.5)	471.9 (374.9; 1706.8)	853.6 (457.8; 1694.1)	0.244
uUSP18	[pg/mL]	102.2 (75.7; 112.1)	256.8 (237.9; 274.1)	303.3 (283.6; 346.0)	-	259.9 (185.0; 327.0)	<0.001
uUSP18/ creat.	[pg/mg]	86.9 (39.0; 227.7)	142.2 (88.6; 249.2)	545.2 (348.6; 723.1)	-	365.8 (155.3; 719.8)	0.65

## Data Availability

The original contributions presented in the study are included in the article, further inquiries can be directed to the corresponding author.
